# MicroRNA and mRNA Signatures in Ischemia Reperfusion Injury in Heart Transplantation

**DOI:** 10.1371/journal.pone.0079805

**Published:** 2013-11-20

**Authors:** Liangyi Zhou, Guoyao Zang, Guangfeng Zhang, Hansong Wang, Xusheng Zhang, Nathan Johnston, Weiping Min, Patrick Luke, Anthony Jevnikar, Aaron Haig, Xiufen Zheng

**Affiliations:** 1 Department of Pathology, Surgery, Medicine, and Microbiology and Immunology, University of Western Ontario, London, Ontario, Canada; 2 Sir Run Shaw Hospital, School of Medicine, Zhejiang University, Hangzhou, China; 3 Lawson Health Research Institute, London Ontario, Canada; 4 Multiple Organ Transplant Program, London Ontario, Canada; University of Toronto, Canada

## Abstract

Ischemia reperfusion (I/R) injury is an unavoidable event occurring during heart transplantation, leading to graft failures and lower long-term survival rate of the recipient. Several studies have demonstrated that microRNAs (miRNAs) are vital regulators of signalling pathways involved in I/R injury. The present study aims to quantify the altered expression levels of miRNA and mRNA upon I/R injury in a mouse heart transplantation model, and to investigate whether these miRNA can regulate genes involved in I/R injury. We performed heterotopic heart transplantation on mouse models to generate heart tissue samples with I/R and non-I/R (control). The expression levels of miRNAs as well as genes were measured in heart grafts by microarray and real time RT-PCR. miRNA alteration in cardiomyocytes exposed to hypoxia was also detected by qRT-PCR. We observed significant alterations in miRNA and gene expression profile after I/R injury. There were 39 miRNAs significantly downregulated and 20 upregulated up to 1.5 fold in heart grafts with I/R injury compared with the grafts without I/R. 48 genes were observed with 3 fold change and p<0.05 and 18 signalling pathways were enriched using Keggs pathway library. Additionally, hypoxia/reperfusion induced primary cardiomyocyte apoptosis and altered miRNA expression profiles. In conclusion, this is the first report on miRNA expression profile for heart transplantation associated with I/R injury. These findings provide us with an insight into the role of miRNA in I/R injury in heart transplantation.

## Introduction

Since the 1970s, heart failure (HF) prevalence has been increasing in the world as a consequence of a decline in coronary artery and cerebrovascular disease mortality [1]. Although there are many treatments available for HF patients, heart transplantation remains the best option for long-term survival for end-stage HF patients [2]. However, this effective treatment for heart failure is severely affected by ischemia reperfusion (I/R) injury occurring during transplantation.

Despite major achievements in heart transplantation, I/R injury is a major contributing factor in graft failure and longer ischemia time has shown to lower the long-term survival rate, especially for older patients [3]. In addition, due to a shortage of donors, physicians are forced to enlarge the donor pool by accepting marginal organs, which include organs from elderly or ill patients, and thus they are more susceptible to I/R injury [3]. Currently, there are no effective treatments against ischemia reperfusion injury. It is important to explore new alternative mechanisms involved in I/R injury during heart transplantation.

miRNAs are endogenous, short, non-coding single-stranded RNAs that are approximately 20 nucleotides in length. miRNAs have emerged as a key player in physiology as well as pathophysiology attributable to its ability to downregulate gene expression through mRNA destabilization/degradation and translation repression by binding onto either 3′ UTR or 5′UTR of the mRNA [4]. Several studies have shown that miRNAs have the ability to regulate the expression profiles of genes in signalling pathways associated with heart diseases, including heart failure, hypertrophy, and ischemia reperfusion injury [5]. Therefore, it is crucial to examine the role of miRNA in heart transplantation and its implications for I/R signalling pathways. In this study, for the first time, we report miRNA expression profiles in I/R injured heart grafts and also investigated mRNA expression profiles that may be affected by miRNAs.

## Materials and Methods

### Animals

Eight weeks old C57BL/6 mice were purchased from Charles River Laboratory (Canada). All procedures involving mouse breeding and surgery were performed according to the guidelines of the Canadian Council of Animal Care and were approved by the Animal Use Subcommittee at the University of Western Ontario, Canada.

### Induction of Cold Ischemia Reperfusion Injury and Heart Transplantation

C57BL/6 mice were anesthetized with ketamine/protophin and injected with 1 ml heparin. Donor hearts were excised from mice and heterotopically implanted into the peritoneal cavity with the donor aorta anastomosed to the recipient abdominal aorta and the pulmonary artery connected to the inferior vena cava. For induction of cold I/R injury, donor hearts were preserved with University of Wisconsin (UW) solution at 4°C for 18 hours before implantation. Meanwhile, the rest of the excised heart was immediately implanted into the recipient to generate non cold ischemia injury heart (non-I/R) as controls. At the endpoint of experiments, mice were sacrificed by injection over dose of ketamine/protophin and heart grafts were harvested for future studies.

### Histological Analysis

At 24 hours post-transplantation, heart grafts were collected from mice and tissue slices were fixed in 10% formalin and processed for histology examination using standard techniques. Formalin tissue was embedded in paraffin and 5 µm sections were stained with hematoxylin and eosin stain (H&E).

### Myeloperoxidase (MPO) Activity

To detect neutrophil infiltration, MPO activity was detected in heart tissues. Paraffin tissue sections were stained with MPO antibody (Santa Cruz, San Diego, CA) following the manufacturer’s instruction. Each slide was examined by light microscopy at 200× magnification.

### Terminal Deoxynucleotidyl Transferase-mediated dUTP Nick End Labelling (TUNEL) Assay

To detect cell apoptosis in heart grafts, TUNEL assay was performed on paraffin tissue sections using an in situ cell death detection kit according to the manufacturer’s instruction (Roche, Mississauga, ON, Canada). Sections were counter-stained with hematoxylin. Each slide was examined by light microscopy at 200× magnification.

### microRNA/RNA Extraction

Fresh heart tissues or primary cardiomyocytes were collected and subjected to the extraction of miRNA/RNA. miRNAs were extracted using a miRNeasy mini Kit (Qiagen, Ontario, Canada). RNA quality was assessed using the Agilent 2100 Bioanalyzer (Agilent Technologies Inc., Palo Alto, CA) and the RNA 6000 Nano kit (Caliper Life Sciences, Mountain View, CA). One microgram of total RNA was used to synthesize cDNA for measuring miRNA expression using miRScript II kit (Qiagen, Ontario, Canada) according to the manufacturer’s manual.

### miRNA Microarray

All sample labeling and GeneChip processing were performed at the London Regional Genomics Centre (Robarts Research Institute, London, Ontario, Canada; http://www.lrgc.ca). One microgram of total RNA was labeled using the Flash Tag Biotin HSR kit from Genisphere (http://www.genisphere.com/array_detection_flashtag_biotin.html). Samples were then hybridized to Affymetrix miRNA 3.0 arrays for 16 hours at 48°C. All washing steps were performed by a GeneChip Fluidics Station 450 and GeneChips were scanned with the GeneChip Scanner 3000 7 G (Affymetrix, Santa Clara, CA) using Command Console v3.2.4. Partek was used to determine ANOVA p-values and fold changes for miRNAs. Species annotations were added and used to filter only those miRNA found in *Mus musculus*. miRNA expression data were submitted to the National Center for Biotechnology Information Gene Expression Omnibus (http://www.ncbi.nlm.nih.gov/geo/) under accession number GSE50885.

### Gene Expression Microarray

Single stranded complementary DNA (sscDNA) was prepared from 200 ng of total RNA as per the Ambion WT Expression Kit for Affymetrix GeneChip Whole Transcript WT Expression Arrays (http://www.ambion.com/techlib/prot/fm_4411973.pdf, Applied Biosystems, Carlsbad, CA). Total RNA was first converted to cDNA, followed by *in vitro* transcription to make cRNA. 5.5 µg of single stranded cDNA was synthesized; end labeled and hybridized, for 16 hours at 45°C, to Mouse Gene 1.0 ST arrays. All washing steps were performed by a GeneChip Fluidics Station 450 and GeneChips were scanned with the GeneChip Scanner 3000 7 G (Affymetrix, Santa Clara, CA) using Command Console v3.2.4. Partek was used to determine ANOVA p-values and fold changes for genes. Gene expression data were submitted to the National Center for Biotechnology Information Gene Expression Omnibus (http://www.ncbi.nlm.nih.gov/geo/) under accession number GSE 50884.

### Quantitative Reverse Transcriptase-polymerase Reaction (q-PCR) for miRNA Expression

miRNAs were extracted from I/R and non-I/R heart tissues using miRNeasy mini Kit (Qiagen, Ontario,Canada), then cDNA were synthesized using miRScript II RT Kit (Qiagen, Ontario). Synthesized cDNA samples were then subjected to real time RT-PCR (Stratagene mx3005P) using miRScript SYBR Green PCR Kit (Qiagen, Ontario) according to instruction of the kit supplier. Thermal profiling for the real time PCR was an initial activation step at 95°C for 10 mins, followed by 40 cycles of: 95°C for 15 s, 55°C for 30 s, 70°C for 30 s. Expression levels between I/R and non-I/R were quantitatively compared using the ΔΔCt method with SNorD6 as the endogenous control for miRNA expression.

### Primary Neonatal Cardiomyocytes Culture

Neonatal ventricular cardiomyocytes were cultured as described by the Feng group [6]. Briefly, ventricular tissues from C57BL/6 mice were isolated and minced within 24 h after birth. Subsequently, cardiomyocytes were dispersed by incubation in a D-Hank’s buffer supplemented with 0.5 mg/ml Liberase (Roche, Worthington Biochemical, Lakewood, NJ) and the cellular suspension was filtered through a polypropylene macro porous filter (mesh opening 105 µm, Spectra Mesh; Spectrum Medical Industries). The suspension was then centrifuged at 200 *g* for 5 min, and the cellular pellet was suspended in medium 199 (M199) with 10% FBS and penicillin-streptomycin (50 µg/ml; GIBCO-BRL). The cellular suspension was pre-plated for 1.5 h at 37°C in 5% CO_2_ to remove any non-cardiomyocytes. Cell density was adjusted to 10^6^ cells/ml using M199 supplemented with 10% FBS, and cells were seeded (5 × 10^5^) in polystyrene, nonpyrogenic24-well culture plates (Becton Dickinson, Franklin Lakes, NJ) precoated with 1% gelatin. Cells were incubated in 5% CO_2_ at 37°C.

### Hypoxia/Reperfusion Model for Cell Culture

Isolated primary cardiomyocytes were cultured in a 24 well plate pre-coated with 1% gelatine for overnight. Culture medium were replaced by 250 µl deoxygenated DMEM medium without FBS and antibiotics and then placed in a chamber with 2% O_2_ at 37°C for 45 min. After hypoxia, cells were reoxygenated by adding 250 µl of complete medium supplemented with 10% FBS and cultured at 5% CO_2,_ 37°C, for 24 h.

### Annexin-V Staining and Flow Cytometry

Cardiomyocytes were harvested using Trypsin and suspended in PBS solution with 2% FBS. Cells were double stained with Annexin-V and PI using Annexin-V kit (ebioscience, San Diego, CA) according to the manufacturer’s instruction. The fluorescence from the stained cells was measured by flow cytometry (BD bioscience, San Jose, CA).

### Potential miRNA Targets

For selected miRNA that were significantly altered by I/R injury in heart transplantation, online computational algorithm analyses (Targetscan and FINDTAR3) were used to predict potential target genes. Based on scientific literature, target genes that are known to be involved in I/R injury pathways were selected for further analysis.

### Gene Expression

Total RNA isolations were performed using the TRIzol reagent (Invitrogen), and cDNA was synthesized using oligo-(dT) primer and reverse transcriptase (Invitrogen) according to the manufacturer’s protocol. Synthesized cDNA samples were then subjected to real time RT-PCR (Stratagene mx3005P and Bio-Rad CFX) with SYBR Green and a final primer concentration of 80 nM. Thermal profiling for the real time PCR was the initial activation step at 95°C for 10 mins, followed by 40 cycles of: 95°C for 30 s, 58°C for 30 s, 72°C for 30 s. Expression levels between I/R and non-I/R were quantitatively compared using the ΔCt method with mouse glyceraldehyde-3-phosphate dehydrogenase (GAPDH) as the endogenous control for target gene expression.

### Western Blotting

Heart tissues were homogenized with RIPA buffer containing 5 µg/ml PMSF as the manufacturer’s instruction (Invitrogen, Life Tech., Ontario, Canada) and centrifuged at 10,000 g for 10 mins. Protein concentration was measured using a Bradford assay (Bio-Rad, Mississauga, Ontario, Canada). 40 µg of total proteins were separated by 12% SDS-PAGE and transferred onto nitrocellulose membranes. After blocking for 1 h in TBS supplemented with 5% milk and 0.1% Tween20, membranes were blotted with rabbit anti mouse polyclone angiopoietin 1 Ab (used at 1∶500 dilution, Abcam. Toronto, ON, Canada) and developed using HRP-conjugated anti-rabbit IgG (1∶5,000 dilution) (Santa Cruz, Dallas, Texas) and enhanced chemiluminescence (Bio-Rad).

### Statistical Analysis

All data are presented as means ± SEM. Statistical comparisons between two groups were performed using student’s t-test and ANOVA was applied for the microarray data. Statistical significance was determined as P<0.05.

## Results

### I/R Induced Cardio Graft Damage

**Figure 1:**
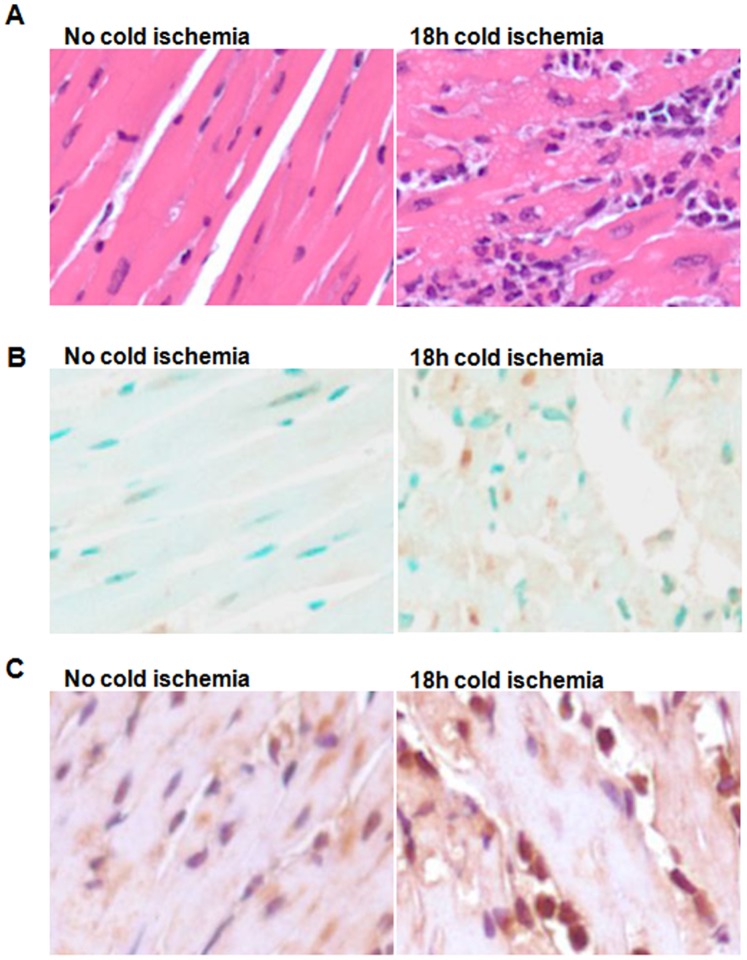
18 h cold ischemia-reperfusion caused I/R injury in heart grafts. Donor hearts were isolated from C57BL/6 mice, infused with UW solution and preserved in UW solution at 4°C for 18 h. After 18 h preservation, donor hearts were implanted into syngeneic recipient C57BL/6 mice. At day 2 after transplantation, the heart grafts were harvested and fixed in 10% formalin. The paraffin heart sections were subjected to HE staining, TUNEL assay, and MPO assay. (**A**) HE staining. (**B**) TUNEL assay for apoptosis. (**C**) MPO assay. Representative images were from experiments.

Currently during organ transplantations, donor organs are preserved using a hypothermal static method to reduce ischemia reperfusion injury. Thus, I/R injury in organ transplantation is usually referred to as cold I/R, differentiating it from that in non-transplanted. Accordingly, we used this hypothermal static method to preserve donor organs for a variety of periods in order to induce cold ischemia-reperfusion injury. Histopathological staining (H&E, apoptosis and neutrophil infiltration) were applied to assess the severity of I/R injury in grafts. We found that 18 h cold ischemia induced I/R injury - supported by results from H&E, TUNEL assay, and MPO assay. H&E staining (Figure 1A) shows disrupted architecture, necrosis, and degeneration in I/R groups compared to the control (non-I/R) group. TUNEL assay (Figure 1B) shows an increased number of apoptotic cells in I/R groups. As shown by MPO assay (Figure 1C), increased myeloperoxidase activity was observed in the I/R group, indicating there are neutrophils infiltrating into grafts.

### I/R Injury Altered miRNA Expression Profiles in Heart Grafts

**Figure 2:**
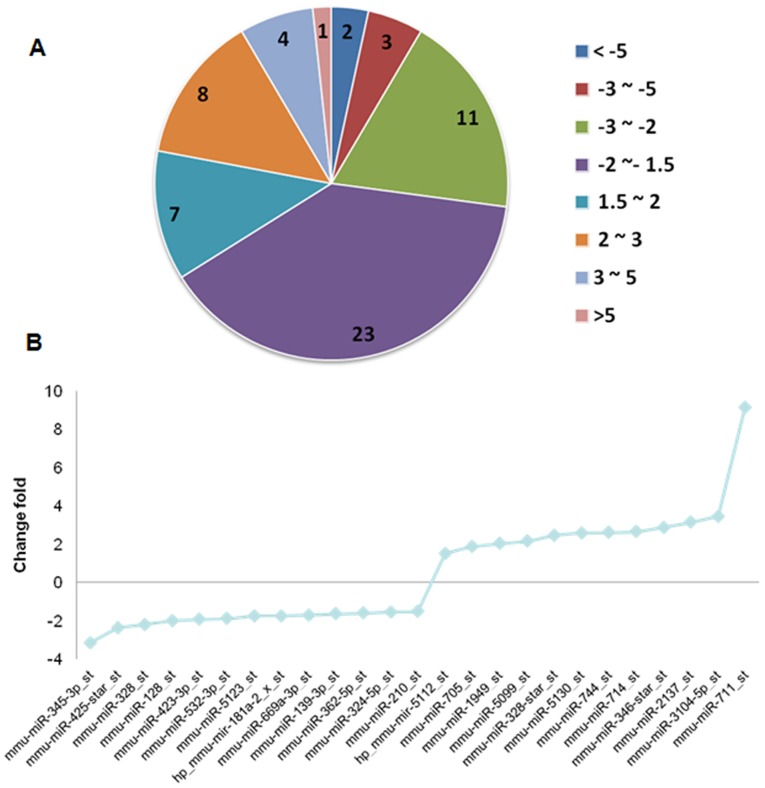
miRNA expression in heart grafts. Donor hearts were treated and transplanted into syngeneic recipient C57BL/6 mice as described in Figure 1. miRNAwere extracted and detected by miRNA array or qPCR at day 2 after transplantation. (**A**) A heat map of miRNA microarrays. (B)A pie graph of miRNA distribution. (**C**). Altered miRNA with its fold change.

In order to characterize the miRNA expression profile that regulates genes involved in I/R injury in heart transplantation, we performed a microarray assay using Affymatrix: GeneChip miRNA 3.0 Array that contains 1111 mouse probe sequences. Heart grafts (n = 3/group) with I/R or with non-I/R (control) were collected to extract miRNA at day 2 post transplantation. Microarray assays showed that miRNA were expressed differentially in heart grafts. A total of 59 miRNA were significantly altered with the criteria of 1.5 fold change with *P*<0.05 (Table 1). Out of the 59 altered miRNAs, 39 were downregulated in heart grafts with I/R injury compared with the grafts without I/R, while 20 miRNA were upregulated (Table 1). As shown in a pie graph of miRNA distribution based on their fold changes in expression (Figure 2A), the majority of altered miRNA (49 out of 59) fell into the range of 1.5 to 3 fold up or downregulation. Only ten miRNAs (five up-regulated and another five down-regulated) displayed over three fold changes between two groups. Each individual altered miRNA with >3 fold change is shown in Figure 2B, indicating that the miRNA changes fit a Poisson distribution. Additionally, signalling pathway enrichment analysis was conducted based on miRNAs with 1.5 fold change and *p*<0.05, 71 signalling pathways were selected (enrichment *P*<0.05) using the Kegg pathway library.

**Table 1:** miRNA altered in heart transplantation.

miRNA	Fold-Change(Exp vs. Control)	SS(Error)	Fold-Change(Exp vs Control)	p-value(Exp vs. ontrol)	
mmu-miR-490-5p_st	−6.24593	1.23515	Down	0.0203434	
mmu-miR-326_st	−5.24883	0.284485	Down	0.00340912	
mmu-miR-490-3p_st	−3.65425	1.19612	Down	0.0477261	
mmu-miR-1843b-5p_st	−3.2084	0.636683	Down	0.0280223	
mmu-miR-345-3p_st	−3.1504	0.691868	Down	0.0325233	
mmu-miR-671-3p_st	−2.92478	0.667747	Down	0.0368904	
mmu-miR-24-1-star_st	−2.85064	0.446847	Down	0.0232897	
mmu-miR-181a-2-star_st	−2.7874	0.358902	Down	0.0183911	
mmu-miR-1943_st	−2.62098	0.223826	Down	0.0113917	
mmu-miR-425-star_st	−2.37163	0.416558	Down	0.0351811	
mmu-miR-181c-star_st	−2.3374	0.0854605	Down	0.00415107	
mmu-miR-185-star_st	−2.32242	0.115686	Down	0.00655456	
mmu-miR-491_st	−2.2317	0.456292	Down	0.0473795	
mmu-miR-328_st	−2.20344	0.412421	Down	0.0434945	
mmu-miR-2183_st	−2.11913	0.0630796	Down	0.00381541	
mmu-miR-3058_st	−2.01364	0.183612	Down	0.0208451	
mmu-miR-128_st	−1.98828	0.0715253	Down	0.00590299	
mmu-miR-874-star_st	−1.977	0.110185	Down	0.0111359	
hp_mmu-mir-181a-2_st	−1.96297	0.169814	Down	0.0207353	
mmu-miR-423-3p_st	−1.93968	0.261325	Down	0.0381555	
mmu-miR-324-3p_st	−1.91486	0.201859	Down	0.0287916	
mmu-miR-532-3p_st	−1.89947	0.18219	Down	0.0260086	
mmu-miR-181a-1-star_st	−1.86983	0.121223	Down	0.0160835	
mmu-miR-467a-star_st	−1.822	0.238043	Down	0.0435423	
mmu-miR-433_st	−1.78743	0.166247	Down	0.0299398	
mmu-miR-351-star_st	−1.75	0.0465557	Down	0.00574918	
mmu-miR-5123_st	−1.74629	0.132341	Down	0.024712	
hp_mmu-mir-181a-2_x_st	−1.74163	0.175927	Down	0.0362254	
mmu-miR-669a-3p_st	−1.69692	0.198551	Down	0.0475402	
mmu-miR-295-star_st	−1.68704	0.195156	Down	0.047832	
mmu-miR-470_st	−1.64899	0.159001	Down	0.0414325	
mmu-miR-139-3p_st	−1.64413	0.0621587	Down	0.012078	
hp_mmu-mir-128-2_x_st	−1.61738	0.0527945	Down	0.0105619	
mmu-miR-362-5p_st	−1.60748	0.155159	Down	0.0457828	
hp_mmu-mir-133a-1_x_st	−1.59055	0.125522	Down	0.0371386	
mmu-miR-190b-star_st	−1.55534	0.030848	Down	0.00627936	
mmu-miR-324-5p_st	−1.53951	0.0934193	Down	0.0306476	
mmu-miR-210_st	−1.52691	0.0256112	Down	0.00543635	
mmu-let-7i-star_st	−1.51651	0.0849839	Down	0.0297218	
hp_mmu-mir-128-2_st	1.5022	0.112589	up	0.045064	
hp_mmu-mir-5112_st	1.50223	0.0196402	up	0.00415309	
hp_mmu-mir-466c-1_x_st	1.62092	0.162286	up	0.0463585	
hp_mmu-mir-101a_x_st	1.66217	0.129606	up	0.0306606	
hp_mmu-mir-466b-4_x_st	1.7585	0.158651	up	0.0303406	
mmu-miR-705_st	1.89157	0.269444	up	0.0436911	
hp_mmu-mir-5130_st	1.96821	0.127422	up	0.013874	
mmu-miR-1949_st	2.02064	0.0914098	up	0.00783574	
mmu-miR-5099_st	2.15647	0.30996	up	0.0324799	
mmu-miR-328-star_st	2.46223	0.335471	up	0.0237448	
mmu-miR-5130_st	2.57367	0.653544	up	0.0492977	
mmu-miR-744_st	2.59709	0.0620819	up	0.00185233	
mmu-miR-714_st	2.65014	0.567341	up	0.0383118	
mmu-miR-346-star_st	2.87902	0.78242	up	0.0466927	
mmu-miR-1896_st	2.91753	0.789354	up	0.0457703	
mmu-miR-1982-star_st	3.12965	0.771611	up	0.0379458	
mmu-miR-2137_st	3.14534	0.670176	up	0.0313255	
mmu-miR-3104-5p_st	3.44502	1.09737	up	0.048136	
mmu-miR-1893_st	4.59124	0.681606	up	0.01495	
mmu-miR-711_st	9.14388	3.24	up	0.0435551	

Note: Exp: grafts with 18 h cold-ischemia and reperfusion; Control: grafts without 18 h cold ischemia.

We further confirmed the expression of miRNA selected from miRNA microarray results by quantitative PCR (Figure S1). The expressions of miR-711, miR-714, miR-744, miR-2137, miR-5130, miR-1892, miR-328, miR-346, miR-5099, and miR-705 were significantly upregulated in I/R injured heart grafts, while miR-490, miR-491, miR-210, miR-362, miR-24, miR-423, miR-128, miR-328, miR -181, and miR-532 were downregulated. According to intensity of fluorescence detected on miRNA microarray and Ct values of qPCR, the expression levels of these miRNA in heart tissues varied to a great extent between each other. For example, miR-2137, miR-5130 and miR-5112 were highly expressed in heart tissues; miR-490, miR-491, miR-181, miR-362, miR-425, and miR-3104 were expressed at quite a low level (Ct value ∼ over 30), whereas 32 out of those 58 altered miRNA were expressed at an extremely low level in hearts and there were almost no Ct value detected by qPCR.

### miRNA Expression Profile was Dynamically Changed in Heart Grafts

**Figure 3:**
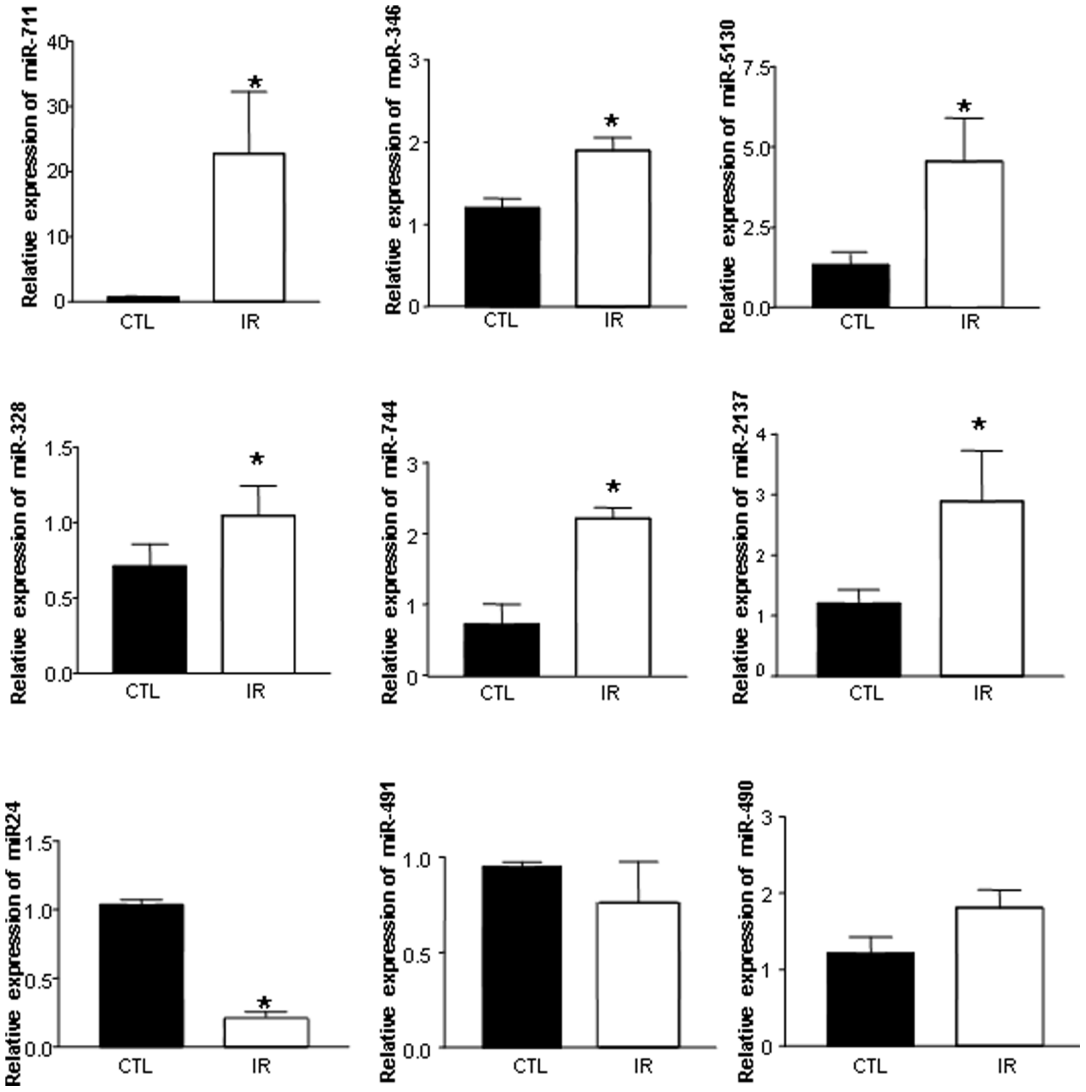
miRNA expression in heart grafts on day 7 post transplantation. Donor hearts were treated and transplanted into syngeneic recipient C57BL/6 mice as described in Figure 1. miRNA were extracted and detected by qPCR on day 7 after transplantation.

Literature has reported that miRNA expression changes dynamically [7]. Accordingly, we investigated whether miRNA expression changes over time in hearts post transplantation. We extracted grafts with I/R and with non-I/R at day 7 after transplantation and detected the expression level of miRNAs (miR-711, miR -714, miR-744, miR -2137, miR -5130, miR -346, miR -490, miR -491, miR -24, and miR -328). As shown in Figure 3, the expressions of miR-711, miR-714, miR-744, miR -2137, miR -5130, miR -346, and miR -328 was still upregulated in the I/R group, but the expression of miR-24 and miR-490 were downregulated, compared to the control group grafts. The expression of miR-491 was slightly but not significantly upregulated in I/R injured grafts. Compared with grafts taken out on day 2 post-transplantation, the expression of miR-2137, miR-714, miR-744, miR -2137, miR -5130, miR -346, and miR -328 were slightly decreased, whilst the expression of miR-711 continued its upregulation in the I/R injured grafts.

### Hypoxia/Reperfusion Incurred Cardiomyocyte Apoptosis and miRNA Expression Change In Vitro

**Figure 4:**
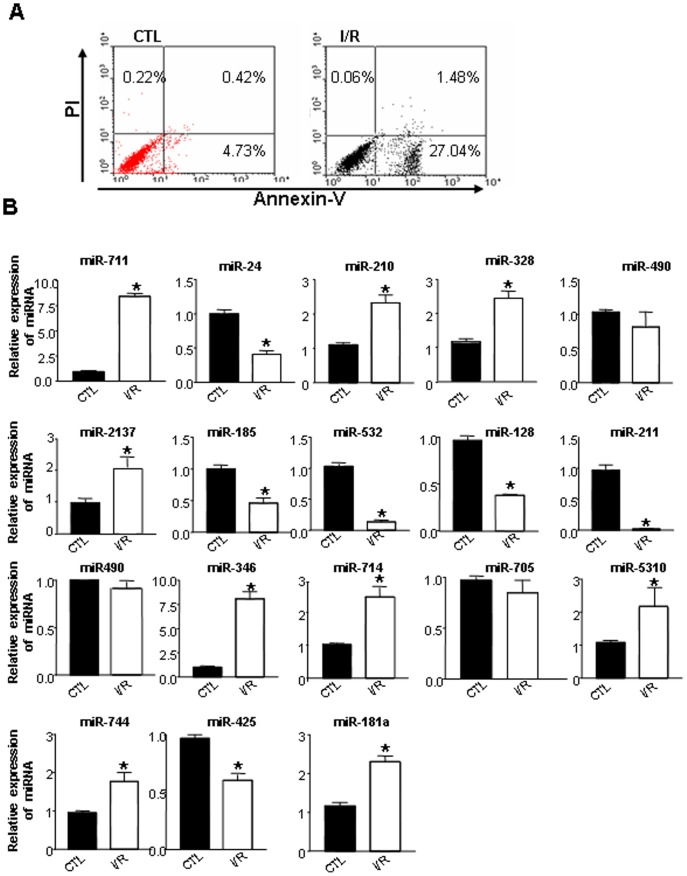
Hypoxia induced apoptosis of primary cardiomyocytes and altered miRNA expression in primary cardiomyocytes. Primary cardiomyocytes were isolated from neonatal C57BL/6 mice cultured in vitro and then subjected to a hypoxia environment. Cell apoptosis was detected by staining with Annexin–V and followed by flow cytometry analysis 24 h after hypoxia. miRNAs were extract from above cells and the expression of miRNA were detected by qPCR.

Cardiomyocyte is a main component of heart tissue. Given that prolonged cold ischemia changed miRNA expression profiles in heart grafts, we proposed to determine what the miRNA profile would be in cardiomyocytes in response to hypoxia/reperfusion stress in vitro and whether it was in line with that from heart tissues in vivo. We isolated and cultured primary cardiomyocytes from neonatal mice and subjected them to hypoxia/reperfusion stress by exposing them to a 2% O_2_ chamber for 45 mins and then reoxygenation for 24 h by incubation of cells in a 5% CO_2_ and 95% O_2_ incubator. We first measured cell apoptosis/death by double staining with Annexin-V and PI to confirm cell injury induced by hypoxia/reperfusion. Results showed that 45 mins hypoxia/reperfusion induced cardiomyocyte apoptosis evidenced by the increased percentage of apoptotic cells (Figure 4A) in hypoxia/reperfusion treated cells as compared with cells without hypoxia stress.

Next, we measured the expression of miRNA in hypoxia/reperfusion -treated cardiomyocytes by qPCR. As compared with cells under normxia, miR-711, miR-714, miR-328, miR-346, miR-210, miR-744, miR-5130, miR-181a and miR-2137 were significantly over-expressed in hypoxia/reperfusion treated cardiomyocytes, while the expression of miR-491, miR-211, miR-532, miR-185, miR-425, miR-128, miR-24 was down-regulated (Figure 4B). There was no significant difference in the expression of miR-490 between the two groups (Figure 4B). As expected, miR-2137, miR-210, miR-5130, and miR-328 were highly expressed in cardiomyocytes, while miR-490, miR-491, and miR-211 were expressed at a low level.

### Prolonged Cold I/R Changed Gene Expression Profile and Signalling Pathways in Heart Grafts

**Figure 5:**
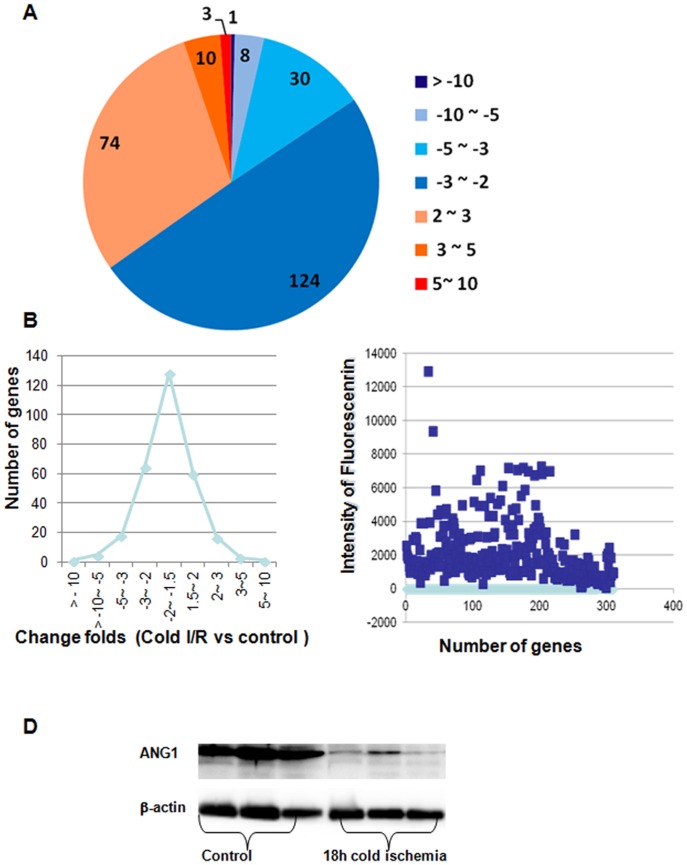
Gene expression in heart grafts detected by microarray assays. Donor hearts were treated and transplanted into syngeneic C57BL/6 recipient mice. At day 2 post transplantation, total RNAs were extracted from grafts and gene expression in grafts were detected by microarray assays. (A) A Poisson distribution of gene expression. (B) Fluorescence intensities of altered genes detected by microarray assays. (C) Altered genes with two fold changes. (D) Angiopoietin 1 expression by Western Blotting.

miRNA functions as a negative regulator of gene expression. Similarly, using microarray assay, we investigated the global gene expression changes in I/R injured heart grafts. Heart grafts with or without prolonged ischemia were harvested at day 2 after implantation and subjected to gene expression microarray assay (Table S1). I/R greatly altered gene expression profiles in heart grafts as shown in a heat map. The expression for the majority of significant altered genes (*P*<0.05) was increased or decreased 1.5–2 fold between two groups and also fits in a Poisson distribution (Figure 5A and 5B). Most of the altered genes were expressed in heart tissue at a moderate level with fluorescence intensities of around 1000–2000 units in microarray (Figure 5C). Given a three-fold change and P<0.05 (up and down) in differential expression as a cut-off, the number of altered genes was reduced to 48; 36 of them were downregulated, and 12 genes were up regulated (Table 2). Among them, Angiopoietin 1(ANG1) decreased to the greatest degree, which was confirmed by the Western blot (Figure 5D).

**Table 2:** Annotated genes regulated in mouse heart transplants.

Gene	p-value	Fold-Change (Exp vs. Control)	Function annotation
Angpt1	0.008529	−19.3306	receptor binding
Asb15	0.0182505	−7.53081	
Gpr22	0.0024004	−6.50553	signal transducer activity
Lrrc10	0.0475174	−5.97936	
Klhl38	0.0359302	−5.9631	
Grm1	0.0210762	−5.86236	PLC activating G-protein coupled glutamate receptor activity
Il15	0.0122715	−5.71981	cytokine activity
Iigp1	0.0378235	−5.64458	nucleotide binding
Art1	0.0219665	−5.48431	NAD+ ADP-ribosyltransferase activity
Ucp3	0.0087787	−4.93265	oxidative phosphorylation uncoupler activity
Adh1	0.0416643	−4.91006	nucleotide binding
Mylk3	0.00744	−4.62612	nucleotide binding
Fam40b	0.0144888	−4.56915	
Csdc2	0.0223918	−4.44072	nucleic acid binding
Tmtc1	0.0141671	−4.27416	
Kcnj3	0.0285984	−4.2726	ion channel activity
Car14	0.0368218	−4.11809	carbonate dehydratase activity
Asb2	0.0086771	−4.10112	
Tmem100	0.0122261	−4.00128	
Slit2	0.0351409	−3.99089	GTPase inhibitor activity
Tnni3k	0.01648	−3.90863	nucleotide binding
Gm4951	0.0269588	−3.81868	no
Lpin1	0.0219087	−3.66845	RNA polymerase II transcription factor binding
Filip1l	0.0181653	−3.62773	
Ppp1r3a	0.0463544	−3.49446	protein serine/threonine phosphatase activity
Hfe2	0.0235755	−3.47549	protein binding
Slc2a4	0.0300101	−3.47473	transporter activity
Ppargc1a	0.0280838	−3.30582	nucleotide binding
Mapk10	0.0245692	−3.29025	nucleotide binding
Slc38a3	0.0377629	−3.24102	L-histidine transmembrane transporter activity
Adck3	0.0251863	−3.2227	nucleotide binding
Pnmt	0.0497843	−3.13239	phenylethanolamine N-methyltransferase activity
Fuca2	0.0231706	−3.10097	catalytic activity
Asb11	0.0384006	−3.07244	
Ube2ql1	0.0294376	−3.04395	nucleotide binding
Hfe	0.0433132	−3.00196	protein binding
Trim16	0.0428384	3.10174	DNA binding
Sfn	0.0394142	3.14658	protein binding
Tnfrsf23	0.0115847	3.26603	receptor activity
Apln	0.0152681	3.36778	receptor binding
Cyr61	0.0336177	3.38045	integrin binding
Pgf	0.0303102	3.53196	growth factor activity
Loxl4	0.0059025	3.71021	protein-lysine 6-oxidase activity
Prnd	0.0159526	4.75449	copper ion binding
Gdf15	0.0093257	4.8554	growth factor activity
Fosl1	0.0168812	5.48284	DNA binding
Plk2	0.0104977	5.50407	nucleotide binding
Crct1	0.01037	7.60055	protein binding

Note: Exp: grafts with 18h cold-ischemia and reperfusion;Control: grafts without 18 h cold ischemia.

**Figure 6:**
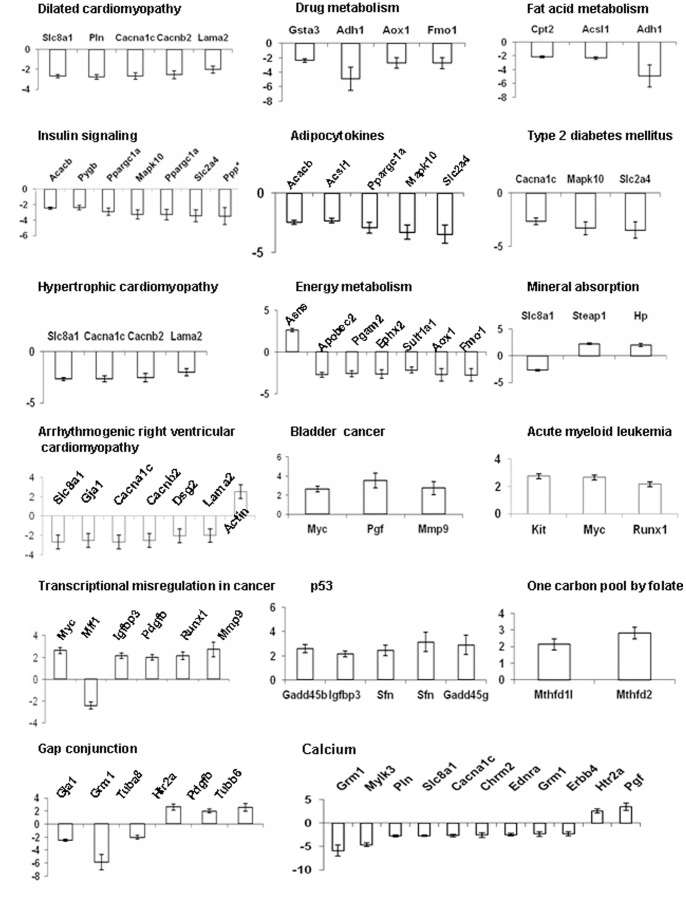
Genes in 18 altered signalling pathways. Gene expression in the heart grafts was detected by microarrays as describe in Figure 5. Signalling pathways were enriched using the Kegg library with 2 fold change and p<0.05.

To clarify which signalling pathways were affected by I/R in heart transplantation, we applied the KEGG library and performed enrichment analysis for microarray data. Eighteen signalling pathways were enriched with the criteria of 2 fold changes and *p*<0.05 (Table 3), which include the Insulin, Tyrosine metabolism, cell cycle, gap junction, calcium, energy metabolism, p53, cardio function associated signalling pathways and some cancer signalling pathways. Less than 10% genes listed on those pathways significantly changed with more than 2 folds. Figure 6 shows the information of these genes from the 18 altered signalling pathways listed on Table 3. Most genes from those pathways were downregulated, except genes in the p53 and one carbon pool by folate, bladder cancer, acute myeloid leukemia and transcriptional misregulation in cancer pathways were upregulated. The Gap junction pathway evenly consisted of both up and downregulated genes.

**Table 3:** Enriched signalling pathways regulated in heart transplantation.

Pathway Name	EnrichmentScore	p-value Enrichment	% genes^a^	# genes^b^	# genes^c^	# genes^d^	Database
Arrhythmogenic right ventricular cardiomyopathy (ARVC)	9.7642	5.75E-05	9.58904	7	89	6829	kegg
Calcium signaling pathway	8.48247	0.000207	5.40541	10	86	6720	kegg
Gap junction	6.56392	0.00141	6.66667	6	90	6811	kegg
Adipocytokine signaling pathway	5.81675	0.002977	6.94444	5	91	6828	kegg
Dilated cardiomyopathy	4.86527	0.00771	5.55556	5	91	6810	kegg
Energy Metabolism	4.60156	0.010036	4	7	89	6727	kegg
p53 signaling pathway	4.22517	0.014623	5.7971	4	92	6830	kegg
Insulin signaling pathway	4.17598	0.01536	4.08163	6	90	6754	kegg
Tyrosine metabolism	3.99802	0.018352	7.31707	3	93	6857	kegg
Bladder cancer	3.81036	0.02214	6.81818	3	93	6854	kegg
Hypertrophic cardiomyopathy(HCM)	3.6154	0.026906	4.81928	4	92	6816	kegg
One carbon pool by folate	3.59657	0.027418	10.5263	2	94	6878	kegg
Mineral absorption	3.52912	0.029331	6.12245	3	93	6849	kegg
Fatty acid metabolism	3.477	0.0309	6	3	93	6848	kegg
Drug metabolism - cytochromeP450	3.46478	0.03128	4.5977	4	92	6812	kegg
Type II diabetes mellitus	3.28047	0.037611	5.55556	3	93	6844	kegg
Transcriptional misregulationin cancer	3.27588	0.037784	3.31492	6	90	6720	kegg
Acute myeloid leukemia	3.01689	0.048953	5	3	93	6838	kegg

note: ^a^% genes in pathway that are present;

b numbers of genes in list and in the pathway;

c numbers of genes in list, not in pathway;

d numbers of genes not in list, not in pathway.

## Discussion

I/R injury is recognized as a primary factor leading to graft dysfunction [8] and graft failure [9], [10]. It is important to understand molecular mechanisms of I/R injury for the development of therapies against I/R injury. The current study, for the first time, reported on the expression profile of miRNA in I/R injured heart grafts in heart transplantation. The findings of our study demonstrate that miR-711, miR-2137 miR-705, miR-5130, miR-346, miR-714, and miR-744 were significantly upregulated (>2 fold change) in I/R injured hearts, while miR-210, miR-490, miR-491, miR-425, miR-423-3p, and miR-532-3p were downregulated. The study also demonstrates that 250 genes and 18 signalling pathways were significantly altered with more than 2 fold changes by I/R injury by cDNA microarray assay.

microRNAs have emerged as a vital regulator in many physiological and pathological pathways. Deregulation of miRNAs associated with different forms of ischemia reperfusion injury [11]. A substantial number of miRNA including miR-1 [12], miR-15 [13], miR-21 [14], [15], miR-24 [16], [17], [18], miR-499 [19], and the miR-17-92 family [20], miR-124 [21], miR-15a/b [22] (2012), miR-93 [23], miR-29 family [24], [25], miR-146a [26], [27], miR-145/451 [28], miR-384-5p [29], miR-424 [30], and miR-494 [31] have been identified in I/R injury. It has been demonstrated that ischemia precondition (IPC) increases cardiac expression of miRNA-1, miRNA-21 and miRNA-24. IPC-regulated miRNA-21 reduces cell apoptosis by repressing the programmed cell death 4 (PDCD4) gene, which results in preventing the heart from I/R injury [32]. It has also been reported that inhibition of miR-15 prevents cardiac ischemia injury in a porcine coronary arterial ligation model [33]. However, in this study we did not observe the above miRNAs change in heart grafts. The reason for this might be that miRNA expression changes dynamically and miRNA expression varies between different models [34], [35].

In this study, we observed that miR-711 was significantly up-regulated both in I/R injured heart grafts and hypoxia/reperfusion treated primary cardiomyocytes. Despite only a few studies of miR-711 have been reported, available data have shown that miR-711 is expressed in many types of cells [36], [37], [38] and is upregulated under different stresses [39], [40]. For example, a chemical palmitate used to induce insulin resistance increases the expression of miR-711 in mouse muscle C2C12 cells [41]. A study has also shown that miR-711 was significantly upregulated in the myocardium with acute myocardial infarction on day 14 post ischemia [42]. Tranter et al reported shows that cardiac ischemic preconditioning (IPC) of the *in vivo* mouse heart results in decreased levels of miR-711 which was dependent on NF-kB, and that miR-711 post-transcriptionally suppresses Hsp70.3 [43]. A more recent study reported that Pioglitazone (an insulin sensitizing drug with cardio protective effect, it attenuates cardiac fibrosis) increased miR-711 levels in myocardial infarction rats and miR-711 directly targeted and downregulated SP1, leading to reduced collagen-I levels [44]. However, the expression level of SP1 and HSP70 was not altered in I/R injured heart tissues in this study (data not shown). It may be attributed to differences in animal model and injury. Predicted by TargetScan and FINDTAR3, Angiopoietin 1 (ANG1) is a putative target of miR-711. Our data also showed that ANG1 was significantly downregulated in I/R injured hearts and hypoxia-treated cardiomyocytes, suggesting ANG1 might be a target of miR-711. Supportively, Lee et al [45] demonstrated that ANG1 can exert cardio protective effects by preventing vascular leakage and cardiomyocyte death by inhibiting activities of Caspase 3 and Caspase 9. Further study on miR-711 function will help us understand the regulatory roles of miR-711.

Additionally, miR-2137, miR-1893, miR-744, miR-705 and miR-714 are highly expressed in heart tissue and cardiomyocytes as well, suggesting that these miRNA are important for cardiomyocytes survival and growth. However, there are no reports available on miR-2137 and 1893 regarding their functions. Performing computational analysis using TargetScan, two conserved genes, retrograde golgi transport homolog (RGP1) and pleckstrin and Sec7 domain containing (Psd), were predicted as targets of miR-2137, while another 144 genes including calmodulin binding transcription activator 1(Camta1) and furry homolog-like (Fryl) were predicted irrespective of site conservation. The data from the gene expression microarray assay showed that the Camta1 gene was significantly downregulated in I/R injured grafts. It has been shown that CAMTA 1 can activate the expression of the anti-proliferative cardiac hormone natriuretic peptide A (NPPA) in the heart and is recognised as a tumor suppressor [46]. Another family member of CAMTA, the transcriptional coactivator CAMTA2 stimulates cardiac growth by opposing class II histone deacetylases, while the loss of CAMTA2 promotes cardiomyocyte hypertrophy [47]. Taken together, the increased expression of miR-2137 may play a role in I/R injury in heart transplantation through regulating CAMTA 1.

miR-705, miR-714 and miR-744 have not been extensively investigated yet. It has been reported that the expression of miR-714 and miR-744 are significantly higher in mice aorta with vascular calcification [48]. miR-744 is known to be expressed in cardiac valves [49] and involved in cancer cell growth and proliferation [50], [51]. Recent studies have shown that miR-744 targets TGF-β and eukaryotic translation elongation factor 1 alpha 2(eEF1A2) which is able to promote cell growth and inhibit apoptosis [52], [53], [54]. Our data showed that eEF1A2 was decreased in I/R injured hearts, implying that there may be a causative relationship between miR-744 and eEF1A2. Predicted by TargetScan, miR-705 has 89 putative targets including Transmembrane BAX inhibitor motif containing 1 (TMBIM1) with conservative sites. TMBIM1 has been well documented as a part of the Bax Inhibitor-1(BI-1) family that has a similar anti-apoptotic function as Bcl-2 and miR-705 induced inactivation of TMBIM1 may be responsible for the increase rate of apoptosis after I/R injury [55]. Although our cDNA microarray data did not show significant changes of TMBIM1 expression at the mRNA level, we can not rule out TMBIM1 as a putative target of miR-705 because that miRNA functions via translation repression as well. Our data also showed that miR-346 was highly expressed in normal heart tissues and upregulated by I/R. There are 90 putative targets of miR-346 predicted by the TargetScan. Most studies on miR-346 have been investigated in a rheumatoid arthritis model [56], [57]. The expression of miR-346 was positively correlated with the severity of ischemic injury in a mouse hepatic ischemia/reperfusion injury model [58]. It has been demonstrated that miR-346 can target receptor-interacting protein 140 (RIP140), TNFα, Leukemia inhibitory factor (LIF), IL18 and antigen peptide transporter 1 (TAP1) [56], [59], [60]. The expression of LIF was decreased in the I/R injured heart grafts (data not shown), indicating miR-346 may negatively regulate LIF.

miR-24 has previously shown a protective effect on I/R injury [16], [17], [18]. We observed a decrease in the expression of miR-24 in the I/R injured heart grafts but it was not significant. However, we did see a significant reduction of miR-24 in hypoxia-treated primary cardiomyocytes, which is consistent with reported literature [16], [17], [18]. Since an entire heart tissue consists of multiple lineages of cells such as cardiomyocyte, endothelial cell, and fibroblast cell, the expression of miR-24 in heart is an accumulation from all cells in the heart, and other cells may buffer the reduction of miR-24 in cardiomyocytes.

The data from gene expression microarray showed that 48 genes (36 downregulated and 12 up-regulated) were significantly changed with more than three fold in prolonged cold I/R. Among them, some genes have been demonstrated to be involved in the growth and function of cardiac cells. For examples, growth of Ankyrin repeat and suppressor of cytokine signaling box-containing protein (ASB) 15 regulates myoblast differentiation [61]; G protein-coupled receptors 22 (GPCRs 22) which are highly expressed in cardiac myocytes and coronary arteries plays an essential role in the regulation of cardiac contractile function and cardiomyocyte apoptosis [27] and Leucine-rich repeat containing 10 (Lrrc10), a cardiac-specific factor, is crucial for proper cardiac development. Deletion of Lrrc10 in mice results in dilated cardiomyopathy [62]. Furthermore, uncoupling protein 3(UCP3) [63], angiopoietin 1 [45] and growth differentiation factor-15 (GDF-15) [64] are anti-apoptotic genes and show protective effects on I/R injury in non-transplantation settings. Therefore, those altered genes might be good targets for the prevention of I/R injury in heart transplantation.

In conclusion, our study demonstrates that ischemia reperfusion injury in heart tissue after transplantation is associated with an altered miRNA profile, which will help us to understand roles of miRNA in I/R injury. In addition, this study provides insights into the mechanisms involved in I/R injury and investigate specific miRNA that regulate genes associated with signalling pathways involved in I/R injury. As miRNAs hold great potential as therapeutic targets, expression levels can be modified to prevent, or revert ischemia reperfusion injury that occurs during heart transplantation - minimizing graft failure and increasing graft long-term survival rate.

## Supporting Information

Figure S1 miRNA expression detected by qPCR. miRNA was extracted from heart grafts at day 2 post transplantation as described in Figure 2. cDNA was synthesized using miRScript II RT Kit. The expression of miRNA was detected by qPCR using SYBRGreen systems.
(TIF)

Table S1 Gene expression with two fold changes and p<0.05 in the heart grafts of I/R injury vs.non-IR, detected by gene expression microarray assays.
(XLS)
