# MicroRNA and mRNA Signatures in Ischemia Reperfusion Injury in Heart Transplantation

**DOI:** 10.1371/journal.pone.0101640

**Published:** 2014-06-24

**Authors:** The PLOS ONE Staff

There is an error in the affiliation for author Guoyao Zang. The correct affiliation #2 is: Sir Run Run Shaw Hospital, School of Medicine, Zhejiang University, Hangzhou, China.
