# Supplementary figures and images for: MicroRNA and mRNA Signatures in Ischemia Reperfusion Injury in Heart Transplantation
Source: PLoS One. 2013 Nov 20;8(11):e79805. doi: 10.1371/journal.pone.0079805 (PMC3835872; doi:10.1371/journal.pone.0079805)

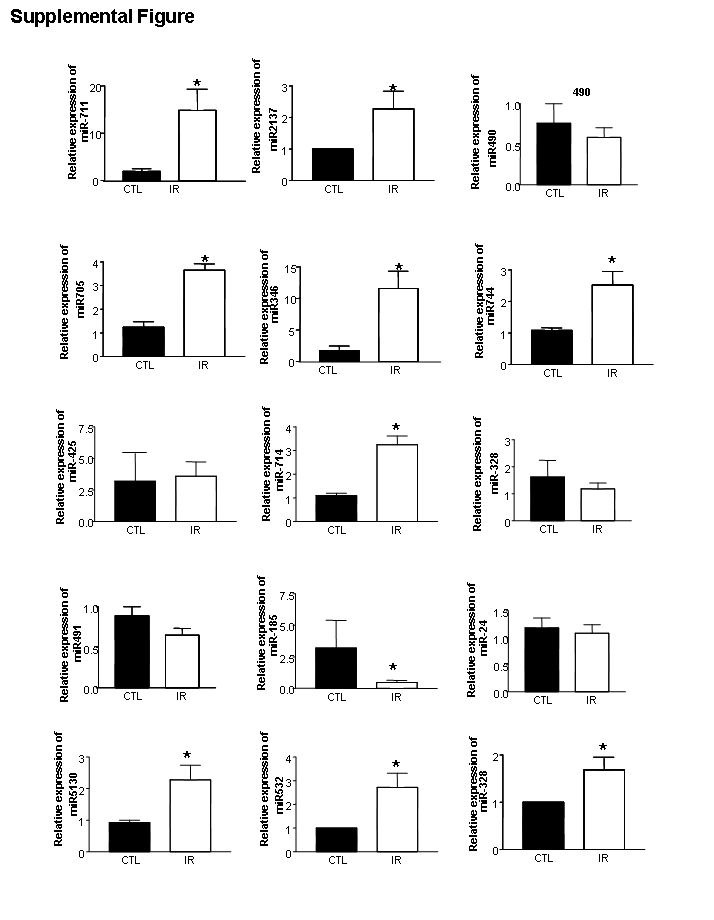

Supplement: Figure S1 — miRNA expression detected by qPCR. miRNA was extracted from heart grafts at day 2 post transplantation as described in Figure 2. cDNA was synthesized using miRScript II RT Kit. The expression of miRNA was detected by qPCR using SYBRGreen systems. (TIF) [file pone.0079805.s001.tif]
